# Pedagogical Progression in Youth Basketball: Impacts on Training Load, Development and Health Outcomes

**DOI:** 10.3390/sports13080265

**Published:** 2025-08-13

**Authors:** Lívia Costa dos Reis Souza, Dilson Borges Ribeiro Júnior, Sergio José Ibáñez, Matheus Neves Rufino Pereira, Gabriel Torres da Silva, Francisco Zacaron Werneck, Maurício Gattás Bara Filho

**Affiliations:** 1 Group of Studies and Research in Basketball (GEPBA), Faculty Physical Education and Sports, Federal University of Juiz de Fora, Juiz de Fora 36036-900, Brazil; liviacreissouza@gmail.com (L.C.d.R.S.); dilsonborges@hotmail.com (D.B.R.J.); matheus.rufino@estudante.ufjf.br (M.N.R.P.); gabriel.torres@estudante.ufjf.br (G.T.d.S.); francisco.zacaron@ufjf.br (F.Z.W.); mgbara1973@gmail.com (M.G.B.F.); 2 Grupo de Optimización del Entrenamiento y el Rendimiento Deportivo (GOERD), Faculdad de Ciencias del Deporte, Universidad de Extremadura, 10003 Caceres, Spain

**Keywords:** SIATE, basketball, sports training

## Abstract

The progression of content during the training and development of young athletes is essential, while considering the developmental stages of the students/athletes. Therefore, it is crucial to monitor training sessions to ensure that content progression is followed and to assess how it is implemented. The aim of this study was to analyze the associations between different male categories of sports development in basketball through pedagogical variables and external loads planned by the coaches. The sample consisted of 148 sessions and 896 tasks, and the SIATE tool was used to observe both the pedagogical variables and the primary external load variables. Significant differences were observed primarily in the U16 category compared to the U12 and U14 categories. In examining the pedagogical variables, three key aspects were highlighted: content type, training methods, and level of opposition. The external load variables were aligned with the pedagogical variables, suggesting a progression of content. This indicates that instruction should follow an order, in which tactical load evolves from the simplest to the most complex, in accordance with the development and training stage of the students/athletes. The analyzed male basketball team demonstrated a content progression focused on the comprehensive development of the student/athlete, encouraging decision-making, and creating a complex, unpredictable, and random environment that closely resembles the dynamics of the real game.

## 1. Introduction

Invasion team sports games can be defined as disciplines in which two teams face each other in the same space and with common objectives, i.e., conquering the target of the opponent and defending their own target [1,2,3,4,5]. Among these modalities, basketball stands out as a team sport of cooperation, invasion and opposition, which has an unpredictable and random character, as well as complex characteristics [5,6]. Based on this complexity, the game demands adaptation from its players, in which decision-making is of great relevance for the final success [7].

Based on this, team sport has become one of the most interesting lines of scientific investigation, focused on the processes of teaching–learning–training (T–L–T) [8,9,10]. Thus, the T–L–T are organized through their internal logic, linked directly to structural element references, as well as functional references [1]. In addition, other factors are the basis for the models and methods, which are linked to the logic of the game: strategy, tactics and technique [11].

Therefore, with a view to developing players, over the years models and methods emerged linked to the T–L–T processes based on the dynamics present in the game [12]. These models are underpinned by interactionist theory and supported by the global functional principle, with the game as the central focus of teaching [6]. In other words, training sessions that use modified game-like tasks with characteristics of the sport, requiring in-depth tactical–technical knowledge, and suitable for the age group of the players [6,13]. In addition, the pedagogical processes must follow the needs of basketball, thus working the student/athlete in an integral form with the possibility of developing the different functions and situations within the game [5].

Further, the process of training coaches and the manner in which training sessions are planned and administered must keep pace with the demands of the game. Therefore encouraging decision-making and the interaction with all the elements and in all its phases, as well as providing an environment that requires skills to solve the challenges proposed by the game itself [10,13]. In addition, the coaches must be vigilant in thinking about different training sessions that meet the developmental stage of the student/athlete [14]. Focusing on cooperative learning and problem-solving in games promotes the development of players who are not only skilled in executing movements, but are also capable of strategic thinking to solve problems, respecting the characteristics of the team sports games [15].

It still highlights the pedagogical progression importance of the content to be stimulated, respecting the developmental stages of the students/athletes. This way, emphasis is placed on the Athlete Development Pathway, a sports development model proposed by the Brazilian Olympic Committee [16], divided into phases that seek to encompass such progression among the categories: (i) experience and play; (ii) play and learn; (iii) learn and train; (iv) train and compete; (v) compete and win; (vi) win and inspire; (vii) inspire and reinvent. Such phases aim to be a desired path of sports development, but it should be emphasized that it must be seen as an open and continuous system. Thus, when analyzing the organization of the contents across different categories, it is essential to identify which stage of the process each one corresponds to, which depends on the sporting and competitive context in which they are embedded.

The pedagogical progression of content during the training and development of the young athlete is necessary, as has been implemented by various basketball schools around the world, notably the International Basketball Federation (FIBA) [17] in conjunction with the World Association of Basketball Coaches (WABC), which proposes a guidance manual for coaches divided through levels, as much for the athletes as for the topics to be developed. The content is not organized by age or category, but rather by levels, starting from the simplest content to the most complex, and subsequently striving to refine what was learned.

In this context, it is essential to perform the monitoring of training sessions, with the perspective of verifying that if the pedagogical progression between contents is respected and in which manner it is performed. As a result, there is an effort in the scientific field to identify valid and well-founded instruments for conducting this type of analysis. In this way, Ibáñez, Feu and Cañadas [18] present the Integral System for the Analysis of Training Tasks (SIATE), an evaluation tool that supports T–L–T and monitors the training sessions planned by coaches. SIATE evaluates variables such as contextual data, coach data, session data, pedagogical variables, organizational variables, external load, internal load and kinematic variables [18].

In view of this, through the pedagogical variables proposed by (SIATE), it is possible to diagnose the tasks, as well as a control and evaluation of the T–L–T processes [19]. Corroborating with the pedagogical variables, the external load variables are able to present the theoretical load of the task and the training session, therefore improving the T–L–T processes and helping the coaches in their decisions [19]. In this way, understanding the pedagogical progression of content may favor the integral development of students/athletes [20].

It becomes evident that it is important to perform the monitoring of the planning for the training sessions by a team of coaches in order to verify in which way the pedagogical progression of the tactical–technical content is carried out. Thus, during the T–L–T processes, following the progress and performance of the coach is a means of controlling and evaluating this pedagogical process, thus contributing to the development of the students/athletes. Therefore, the aim of this study was to analyze the associations among different categories of basketball sports training and the pedagogical variables and primary external loads planned by the coaches.

## 2. Materials and Methods

The design of this study falls within the scope of empirical studies using quantitative and descriptive methodology [21]. It studies the progression of content in the categories of a male basketball team, without the intervention of a researcher and based on planned training sessions.

### 2.1. Participants and Sample

Three coaches were observed with an average of 1.34 ± 0.58 years of experience in youth basketball training, all of whom have an undergraduate degree in Physical Education, and only one of whom holds the Level I coaching qualification from the IBB (Institute of Basketball Brazil). The teams observed were the U16 (n = 20 students/athletes), U14 (n = 27 students/athletes) and U12 (n = 20 students/athletes) male teams, which compete in regional competitions appropriate for their level of development. The research was approved by the Ethics Committee, CAAE: 74357823.4.0000.5147.

The sample consisted of 148 sessions and 896 tasks, none of the tasks were excluded. Comprising 54 training sessions of the U16 (n = 355), training three times a week for a total of five h, 57 of the U14 (n = 351), training three times a week for a total of four and a half hours a week and finally 37 of the U12 (n = 190), training twice a week for two and a half hours a week. These sessions take place during the first half of the season, involving the preparation phase and the early stages of competitions.

### 2.2. Instrument and Study Variables

For this research, SIATE (“Integral System for the Analysis of Training Tasks”) [18], was utilized as the instrument, therefore the variables of the instrument itself were used with some adaptations, these being pedagogical variables (Table 1) and external load variables (Table 2). The adaptations made to the SIATE categories followed methodological precedents from previous studies that also applied adjustments to the instrument [19,22]. These adaptations sought to contextualize the analysis to the specificities of the present study while preserving coherence with the original logic of the tool.

### 2.3. Procedures

The training plans were provided by the coaches prior to their execution, using a previously structured training model. A single researcher was responsible for analyzing and recording the planned pedagogical variables and external load variables. To this end, the Excel platform (Version 2507) was used for task entry and coding. As the analysis was conducted by a single evaluator, inter-rater reliability verification was not necessary.

### 2.4. Data Analysis

A descriptive analysis was carried out on the study variables. Next, in order to identify the association between the age categories (U12, U14 and U16) to observe the pedagogical progression based on the escalation of athlete categories, the pedagogical variables and the primary external load variables planned by the coaches for each training task/session were used, the Chi-square (X2) and V of Crammer [23] were applied.

The association between the independent variables (category) and the dependent variables (Pedagogical and primary external loads) was interpreted using the Corrected Typified Residuals (CTR) (CTR > |1.96|) of the contingency tables. The impact of this association between the variables was analyzed according to the proposal by Crewson [24]. The value of *p* < 0.05 was applied to verify the significance of the results [25]. The data was analyzed using the statistical software SPSS version 26.0 for Mac.

## 3. Results

The results were obtained from the frequencies with which the coaches utilized specific tasks during the training sessions, organized by age categories. Table 3 presents the Corrected Typified Residuals (CTR), which highlight the associations between the pedagogical variables and the different age categories.

In relation to the physical-motor variable, significant differences were observed between U12 (CTR = 4.7) and U16 (CTR = 4.2) in comparison with the U14 (CTR = −8.1). In situations without opposition, there was variation between U14 (CTR = 2.13) and U16 (CTR = 3.8). In contexts of individual equality, the most outstanding were U12 (CTR = 4.1) and U14 (CTR = 3.3), both diverging from U16 (CTR = −6.7). As for the group equality, the difference was between U12 (CTR = −3.2) and U14 (CTR = 2.3). Finally, in the context of collective equality (Full Game), the U16 (CTR = 2.4) differed from the other categories.

Regarding the game phase, in the attacking phase, there was a difference between U12 (CTR = 5.8) and U14 (CTR = 2.7) in relation to U16 (CTR = −7.6). In the defense phase, the U12 (CTR = −4.0) and U14 (CTR = 2.0) exhibited significant variations. In the mixed phase, U12 (CTR = −6.5) differed from U16 (CTR= 7.0). In the offensive transition, there was a difference between U14 (CTR = 5.3) and U16 (CTR = −6.8). With respect to the content type, U16 (CTR = 8.1) showed differences in comparison with U12 (CTR = −3.5) and U14 (CTR = −5.1) in strategic content. In group tactical content, the divergence was between U14 (CTR = 5.7) and U16 (CTR = −4.7). In individual tactical content, there was significance between U12 (CTR = 3.5) and U14 (CTR = 6.6) in relation to U16 (CTR = −9.5). In the technical content, U16 (CTR = 7.8) differed from U12 (CTR = −3.2) and U14 (CTR = −5.2).

In respect to the teaching method, the analytical and Walk-Through tasks displayed significant differences from U16 (CTR = 7.5; 7.7, respectively) to the U12 (CTR = −3.1; −3.5, respectively) and U14 (CTR = −5.0; −4.8, respectively). In small-sided games I, the difference was between U14 (CTR = 10.0) and U16 (CTR = −11.4), while in small-sided games II, the variation was between U12 (CTR = −3.8) and U14 (CTR = 3.8). Finally, in the pre-sports games, U16 (CTR = 2.1) demonstrated variation, as did the U14 (CTR = −2.7) in the deliberate game.

At the opposition level, the tasks with opposition presented a difference between the U14 (CTR = 11.7) and U16 (CTR = −12.1). In situations without opposition, the difference was between U12 (CTR = −4.2) and U14 (CTR = −6.9) in comparison with U16 (CTR = 10.5). Finally, in modulated opposition, U16 (CTR = 2.5) was different from the other categories.

Table 4 presents the correlation (CTR) of the coefficients evidencing the associations between the primary external load variables and the different age categories. These results ratify the outcomes revealed by the pedagogical variables, due to the fact that they are complementary. However, the difference in equality in opposition degree stands out, since U12 (CTR = −3.5) and U16 (CTR = −2.5) is divergent from the U14 (CTR = 7.6). Referring to task density, the oldest category, U16, manifests a greater intensity in relation to the other categories, and there is also a difference between U14 and U12.

In addition, in terms of the simultaneous performers, there is a participation attenuation from the youngest categories, U12 and U14, to the oldest category, U16. When it comes to competitive load, the appreciation of the technical gesture is more frequent in U16 (CTR = 11.1), than in U12 (CTR = −3.6) and U14 (CTR = −8.1), and the same happens for the counting of results without opposition. In opposition without accounting, the difference is from U14 (CTR = 17.2) to U16 (CTR = −16.8). Finally, in reduced and collective opposition with counting, the significance is from U16 (CTR = 5.1; 4.5, respectively) to U14 (CTR = −6.5; −4.8, respectively).

Regarding the game space utilized, the categories present a concordance as to whether the entire court is used, with and without repetition, but U16 uses the dimensions of the court less when compared with the other categories. Lastly, the cognitive involvement in 1×1 to 2×2 situations, in the U12 (CTR = 4.1) and U14 (CTR = 10.7), there is a higher frequency than in U16 (CTR = −14.1), while in 1×0 U16 (RTC = 7.5) is significant as opposed to U12 (CTR = −4.3) and U14 (CTR = −3.9). In 3×0 to 3×3, the difference is between U12 (CTR = −2.2) to the U16 (CTR = 3.2), in 4×0 to 4×4, the difference is between U12 (CTR = −3.6) to the U14 (CTR =3.0), and finally in 5×0 to 5×5, the significance is between U14 (CTR = −3.7) to the U16 (CTR = 4.2).

The inferential results in Table 5 show statistically significant differences (*p* ˂ 0.5) in all the pedagogical variables and primary external load variables.

## 4. Discussion

The objective of this study was to investigate the associations between different sports training categories in basketball and the pedagogical variables, as well as the primary external loads programmed by the coaches. Since there is a need to expand knowledge of T–L–T processes, between applied practice and research-based knowledge is fundamental for the research that is applied in practice [14].

The studies help with how to train and show us how to use pedagogical variables in training, thus collaborating with the organization, systematization, application and evaluation. Furthermore, understanding the sports training and its suitability in the development process becomes even more important in the early stages of training, since it can affect future results [10,26].

In view of this, the results presented in this study mainly pointed to significant differences if we compare the U16 category in relation to the U12 and U14 categories. Similarly to the study by Cañadas et al. [14], the research demonstrated a proximity in the categories studied, and therefore the fact that there were no great differences may be conditioned by the age ranges not being too far apart. Therefore, it is important and necessary for future studies to analyze more categories and with more differences in age groups.

Moreover, it is possible to think in a classification of the categories following the Athlete Development Pathway [16], whereby the U12 category is in the play and learn phase, while the U14 is in the transition from this to learning and training, while the U16 is in the evolution to training and competing. In addition, there is a common concern among several countries with long-term development, hence when the contents are progressive and well distributed by age, they can avoid abandonment, injuries and psychological exhaustion [27]. In Brazil, the Institute Basketball Brazil (IBB) developed a suggestion for the progression of contents related to the sports development in basketball.

Therefore, when we analyze the results presented, the significant differences in the U16 category in relation to the U12 and U14 categories are present in the strategic and technical content, and the walk-through teaching method. As presented, the most advanced category, U16, uses these strategies because there is a need to add more content for the students/athletes, since they have already been through the other two stages. Additionally, when they move up a category, the competitive demands increase, not only in the perspective of results, but also in the number of games and competitions. This means they need technical and strategic reinforcement, as well as more instruments to enable them to deal better with the adversities of the game, thus justifying the strategic content. This occurs since at each formative stage, the contents are transmitted in a specific way, utilizing appropriate teaching methods for each stage of development [28]. Thereby, greater use of individual tactical work by the younger categories is coherent, due to the fact that working on tactics from the beginning is of great importance, as it generates a player who is cognitively involved, creative and autonomous [29]. Additionally, the younger categories exhibit a need for greater development of the game elements at the start of their trajectory in the modality, increasing the time of contact with the ball for the athletes. Also, in terms of pedagogical variables, when specifying or clarifying the levels opposition, it was found that there were a greater number of tasks with opposition in the first two categories with respect to the last. According to Mancha-Triguero et al. [30], the situations with opposition should be frequent, in order to develop the decision-making skills of the students/athletes, as well as increase the training load, thus bringing them closer to the game itself.

In the primary external load variables, the results are in line with those of the pedagogical variables and reinforce a possible progression of content, as it should follow an order, going from the simplest to the most complex, according to the training stages and the development of the students/athletes [13]. Therefore, it is worth emphasizing that the pedagogical variables directly influence the load; therefore, using tasks with greater opposition and realistic game situations (such as 3×3, 5×5) increases the physical and cognitive demand [31].

Following a progression, the competitive load and the cognitive involvement gradually increase, since the more advanced category needs a high intensity and a greater cognitive involvement than the beginner category. This can be related to the fact that reduced situations (1×1 to 3×3) allow the student/athlete to have more time to practice, improve and stimulate their tactical perception [31]. However, it can be seen that at U12 and U14 in the primary variable of external load, referring to the game space, the court spaces are used more frequently, making the intensity high for the category, and thereby favoring development [32].

Thus, when the pedagogical intention is based on the global-functional principle, through games that stimulate decision-making, there is a need to verify the pedagogical progression, as Maricone et al. [5] did with the proposal of the Spanish Basketball Federation (SBF) for initiation and training in basketball, which proposes the following sequence of content: divide and pass, pass and cut, post play, indirect and direct blocks, and mismatches, thus favoring a complete training of the student/athlete, as well as allowing a game with autonomous and cooperative decision-making.

In Brazil, with regard to the pedagogical progression, level 1 of the Brazil Basketball Institute [33] states that in the U12 category, simple play with the ball should be worked on and games without the ball should be introduced, such as dividing and passing, as well as passing and cutting. In the U13, the defensive problems should be presented and the game in the interior space (triangle game and indirect cutback) should be introduced, while in U14, there is a preparation for the entry into performance sport, where more complex tactical concepts will be introduced and played in a more specialized manner.

The team analyzed presents a logical progression of the contents, although it is possible to identify possible suggestions. In the U14 category, the cognitive implication could be more gradative and not with a greater focus on games up to 2 × 2, because the development stage is to learn and train, and in U16 the types of contents and means of training could be more focused on games, in order to stimulate greater training in decision-making.

Just as the progression of contents is important in the development process of the student/athlete, the proper management of training loads is also essential to prevent injuries and enhance sports performance [34]. The progressive manipulation of structural elements (such as space, number of players, and rules) and pedagogical aspects of the tasks allows coaches to control the training load and facilitate the students/athletes adaptation. This approach generates varied physical stimuli and contributes to injury prevention [35].

Likewise, the appropriate progression of both contents and training loads is fundamental in promoting the long-term engagement of students/athletes in sports. The way coaches plan and distribute contents throughout training sessions and age categories can reduce the occurrence of dropout, injury, and psychological burnout, thus increasing the likelihood of continued sports participation [27]. Additionally, task diversity plays a decisive role in maintaining variety, which helps lower the risk of early dropout and promotes healthy sports participation [9].

Finally, the results offer relevant practical applications, as the identification of a logical progression in tactical–technical content may guide the elaboration of training plans which are more suited to the development stage of the athletes. In addition, it is important to highlight that the use of small-sided games and different game situations, such as 2v2 and 3v3, constitutes an effective didactic tool to promote decision-making and cognitive development in student/athletes in training [9].

However, the limited number of categories analyzed (U12, U14 and U16) may have reduced the differences found; furthermore, the analysis focused only on the first half of the season did not consider possible pedagogical adjustments throughout the year. Finally, the exclusive selection of male participants limits the results, indicating the need for future studies to include a broader range of categories and athletes of both sexes for a more comprehensive understanding of the developmental process.

## 5. Conclusions

The male basketball team analyzed presents a coherent progression of contents, aligned with the integral development of the student/athlete. This pedagogical organization is founded on the global–functional principle, which prioritizes the use of reduced games as a central teaching tool. Therefore, favoring the decision-making in complex, unpredictable and dynamic contexts, approximating the training environment to the real demands of the game and potentializing tactical–technical learning from the earliest categories.

## Figures and Tables

**Table 1:** Pedagogical Variables.

Game Situation	Game Phase	Content Type	Taeching Method	Opposition Level
Physical-Motor	Attack	Strategic	Analytical	Physical-Motor
Without Opposition	Defense	Collective Tactical	Walk-Through	With Opposition
Numerical Superiority	Mixed	Group Tactical	Small-Sided Games I	Without Opposition
Numerical Inferiority	Offensive Transition	Individual Tactical	Small-Sided Games II	Modulated Opposition
Individual Equality	Defensive Transition	Technical	Collective	Opposition Stopped
Group Individual	Physical-Motor	Physical-Motor	Pre-Sports Games	
Collective Equality (Full Games)			Deliberate Game	
Momentary Superiority				

**Table 2:** Primary External Load Variables.

Opposition Degree	Task Density	Simultaneous Performers %	Competitive Load	Game Space	Cognitive Involvement
Physical-motor	Physical-motor	1–20%	Physical-motor	Physical-motor	Physical-motor
Without opposition	Smooth or trot	21–35%	Appreciation of the technical gesture	Free throw/Static	1×0
Superiority +3	Smooth and Continuous	36–55%	Counting Of Results Without Opposition	1/4 Court	1×1; 2×0; 2×1; and 2×2
Superiority +2	Intensity With Rest	56–80%	Opposition Without Accounting	1/2 Court	3×0 to 3×3
Superiority +1	Intensity Without Rest	81–100%	Group Opposition With Counting	Court Without Repetition	4×0 to 4×4
Equality	High Intensity		Collective Opposition With Counting	Court With Repetition	5×0 to 5×5

**Table 3:** Descriptive Result and CTR of Pedagogical Variables by Category.

		Categories								
Variables		U12		U14				U16		
		n	%	CTR	n	%	CTR	n	%	CTR
Game Situation	Physical-Motor	37	19.5	4.7 *	0	0.0	−8.1 *	55	15.5	4.2 *
	Without Opposition	13	6.8	−1.7	20	5.7	2.13 *	127	33.0	3.8 *
	Numerical Superiority	53	27.9	1.06	104	26.9	0.88	30	8.5	**−** 1.77
	Numerical Inferiority	0	0.0	0.0	0	0.0	0.0	0	0.0	0.0
	Individual Equality	35	18.4	4.1 *	51	14.5	3.3 *	7	2.0	−6.7 *
	Group Individual	10	5.3	−3.2 *	102	29.0	2.3*	76	21.5	0.4
	Collective Equality	24	12.6	−0.6	40	11.4	−1.8	62	17.5	2.4 *
	Momentary Superiority	18	9.5	0.1	44	12.5	0.94	7	2.0	−1.06
Game Phase	Attack	108	56.8	5.8 *	155	44.2	2.7 *	83	23.4	−7.6 *
	Defense	1	0.5	−4.0 *	33	9.4	2.0 *	31	8.7	1.4
	Mixed	7	3.7	−6.5 *	64	18.2	−1.5	115	32.4	7.0*
	Offensive Transition	37	19.5	1.8	82	23.4	5.3 *	19	5.4	−6.8 *
	Defensive Transition	0	0.0	−0.9	1	0.3	−0.2	2	0.6	1.0
	Physical-Motor	37	19.5	0.7	16	4.6	−8.2 *	105	29.6	7.6 *
Content Type	Strategic	0	0.0	−3.5 *	1	0.3	−5.1 *	43	12.1	8.1 *
	Collective Tactical	35	18.4	**−** 0.9	75	21.4	0.4	75	21.1	0.3
	Group Tactical	57	30.0	**−** 1.2	157	44.7	5.7 *	87	24.5	−4.7 *
	Individual Tactical	49	25.8	3.5 *	97	27.6	6.6 *	9	2.5	−9.5 *
	Technical	12	6.3	−3.2 *	21	6.0	−5.2 *	86	24.2	7.8 *
	Physical-Motor	37	19.5	4.7 *	0	0.0	−8.1 *	55	15.5	4.2 *
Teaching Method	Physical-Motor	37	19.5	4.7 *	0	0.0	−8.1 *	55	15.5	4.2 *
	Analytical	12	6.3	−3.1 *	21	6.0	−5.0 *	83	23.4	7.5 *
	Walk-Through	0	0.0	−3.5 *	2	0.6	−4.8 *	41	11.5	7.7 *
	Small-Sided Games I	107	56.3	1.7	252	71.8	10.0 *	97	27.3	−11.4 *
	Small-Sided Games II	0	0.0	−3.8 *	33	9.4	3.8 *	18	5.1	−0.7
	Collective	30	15.8	0.9	43	12.3	−1.1	51	14.4	0.4
	Pre-Sports Games	0	0.0	−0.9	0	0.0	−1.4	3	0.8	2.1 *
	Deliberate Game	4	2.1	1.2	0	0.0	−2.7 *	7	2.0	1.6 *
Opposition Level	Physical-Motor	37	19.5	4.6 *	0	0.0	−8.2 *	56	15.8	4.3 *
	With Opposition	141	74.2	0.5	331	94.3	11.7 *	179	50.4	−12.1 *
	Without Opposition	12	6.3	−4.2 *	20	5.7	−6.9 *	115	32.4	10.5 *
	Modulated Opposition	0	0.0	−1.0	0	0.0	−1.6	4	1.1	2.5 *
	Opposition Stopped	0	0.0	−0.5	0	0.0	−0.8	1	0.3	1.2

* significant for CTR > |1.96|.

**Table 4:** Descriptive Results and CTR of Primary External Load Variables by Category.

Variables		Categories								
		U12			Variables		U12			
		n	%	n	n	n	RTC	n	%	n
Opposition Degree	Physical-Motor	37	19.5	4.6 *	0	0.0	−8.2 *	57	16.1	3.2 *
	Without Opposition	11	5.8	−5.0 *	29	8.3	−6.2 *	124	34.9	7.3 *
	Superiority +2	0	0.0	−0.5	1	0.3	1.2	0	0.0	−0.6 *
	Superiority +1	69	36.3	5.7 *	91	25.9	2.7 *	31	8.7	−5.1 *
	Equality	73	38.4	−3.5 *	230	65.5	7.6 *	143	40.3	−2.5 *
Task Density	Physical-motor	37	19.5	4.6 *	0	0.0	−8.2 *	57	16.1	4.4 *
	Smooth or trot	0	0.0	−2.4 *	0	0.0	−3.7 *	21	5.9	5.7 *
	Smooth And Continuous	11	5.8	−2.1 *	3	0.9	−7.2 *	73	20.6	8.9 *
	Intensity With Rest	133	70.0	11.7 *	90	25.6	−4.4 *	84	23.7	−5.4 *
	Intensity Without Rest	8	4.2	−7.0 *	191	54.4	17.8 *	8	2.3	−12.0 *
	High Intensity	1	0.5	−7.6 *	67	19.1	−0.6	112	31.5	6.9 *
Simultaneous performers %	1–20%	2	1.1	−2.2 *	0	0.0	−4.7 *	31	8.7	6.5 *
	21–35%	5	2.6	−3.8 *	30	8.5	−2.8 *	76	21.4	6.6 *
	36–55%	17	8.9	−2.0 *	27	7.7	−4.6 *	86	24.2	6.7 *
	56–80%	8	4.2	−2.6 *	57	16.2	4.7 *	27	7.6	−2.1 *
	81–100%	158	83.2	4.3 *	237	67.5	4.1 *	135	38.0	−10.4 *
Competitive Load	Physical-motor	37	19.5	4.6 *	0	0.0	−8.2 *	57	16.1	4.4 *
	Appreciation of the technical gesture	6	3.2	−3.6 *	0	0.0	−8.1 *	85	23.9	11.1 *
	Counting Of Results Without Opposition	4	2.1	−2.8 *	15	4.3	−2.2 *	40	11.3	4.6 *
	Opposition Without Accounting	108	56.8	−0.4	328	93.4	17.2 *	85	23.9	−16.8 *
	Group Opposition With Counting	26	13.7	1.7	7	2.0	−6.5 *	59	16.6	5.1 *
	Collective Opposition With Counting	9	4.7	0.3	1	0.3	−4.8 *	29	8.2	4.5 *
Game Space	Physical-motor	37	19.5	4.6 *	0	0.0	−8.2 *	57	16.1	4.4 *
	Free throw/Static	1	0.5	−1.6	0	0.0	−3.3 *	16	4.5	4.6 *
	1/4 Court	37	19.5	−2.8 *	186	53.0	13.6 *	25	7.0	−11.2 *
	1/2 Court	55	28.9	2.6 *	31	8.8	−7.7 *	112	31.5	5.5 *
	Court Without Repetition	8	4.2	−1.0	22	6.3	0.6	21	5.9	0.2
	Court With Repetition	52	27.4	−1.6	112	31.9	−0.1	124	34.9	1.4
Cognitive Involvement	Physical-motor	37	19.5	4.6 *	0	0.0	−8.2 *	57	16.1	4.4 *
	1×0	8	4.2	−4.3 *	28	8.0	−3.9 *	86	24.2	7.5 *
	1×1; 2×0; 2×1; e 2×2	92	48.4	4.1 *	200	57.0	10.7 *	28	7.9	−14.1 *
	3×0 a 3×3	19	10.0	−2.2 *	45	12.8	−1.4	70	19.7	3.2 *
	4×0 a 4×4	1	0.5	−3.6 *	32	9.1	3.0 *	22	6.2	0.1
	5×0 a 5×5	33	17.4	−0.7	46	13.1	−3.7 *	92	25.9	4.2 *

* significant for CTR > |1.96|.

**Table 5:** Association between planned training tasks of categories U12, U14 and U16 and pedagogical variables and external loads Primárias.

Categories		X^2^	*p*	V of Cramer	*p*	T.E. Crewson
Pedagogical Variables	Game Situation	396,292	0.001 *	0.470	0.001 *	Moderate
	Game Phase	210,641	0.001 *	0.343	0.001 *	Moderate
	Content Type	273,636	0.001 *	0.391	0.001 *	Moderate
	Teaching Method	268,946	0.001 *	0.387	0.001 *	Moderate
	Opposition Level	207,633	0.001 *	0.349	0.001 *	Moderate
Primary External Load Variables	Opposition Degree	231,443	0.001 *	0.359	0.001 *	Moderate
	Task Density	536,119	0.001 *	0.561	0.001 *	High
	Simultaneous performers %	192,435	0.001 *	0.328	0.001 *	Moderate
	Competitive Load	405,126	0.001 *	0.475	0.001 *	Moderate
	Game Space	273,214	0.001 *	0.390	0.001 *	Moderate
	Cognitive Involvement	281,754	0.001 *	0.397	0.001 *	Moderate

* significant for *p* < 0.05.

## Data Availability

Data are unavailable due to privacy restrictions.
